# Loss of *Mrap2* is associated with *Sim1* deficiency and increased circulating cholesterol

**DOI:** 10.1530/JOE-16-0057

**Published:** 2016-07-01

**Authors:** T V Novoselova, R Larder, D Rimmington, C Lelliott, E H Wynn, R J Gorrigan, P H Tate, L Guasti, The Sanger Mouse Genetics Project, S O’Rahilly, A J L Clark, D W Logan, A P Coll, L F Chan

**Affiliations:** 1 Centre for EndocrinologyQueen Mary University of London, William Harvey Research Institute, Barts and the London School of Medicine and Dentistry, Charterhouse Square, London, UK; 2 University of Cambridge Metabolic Research LaboratoriesMRC Metabolic Disease Unit, Wellcome Trust-MRC Institute of Metabolic Science and NIHR Cambridge Biomedical Research Centre, Addenbrooke’s Hospital, Cambridge, UK; 3 Wellcome Trust Sanger InstituteWellcome Trust Genome Campus, Hinxton, Cambridge, UK

**Keywords:** obesity, melanocortin, accessory protein, metabolism, MC4R, MRAP2, SIM1, OXT, AVP, CRH, TRH

## Abstract

Melanocortin receptor accessory protein 2 (MRAP2) is a transmembrane accessory protein predominantly expressed in the brain. Both global and brain-specific deletion of *Mrap2* in mice results in severe obesity. Loss-of-function *MRAP2* mutations have also been associated with obesity in humans. Although MRAP2 has been shown to interact with MC4R, a G protein-coupled receptor with an established role in energy homeostasis, appetite regulation and lipid metabolism, the mechanisms through which loss of *MRAP2* causes obesity remains uncertain. In this study, we used two independently derived lines of *Mrap2* deficient mice (*Mrap2^tm1a/tm1a^*) to further study the role of *Mrap2* in the regulation of energy balance and peripheral lipid metabolism. *Mrap2^tm1a/tm1a^* mice have a significant increase in body weight, with increased fat and lean mass, but without detectable changes in food intake or energy expenditure. Transcriptomic analysis showed significantly decreased expression of *Sim1*, *Trh*, *Oxt* and *Crh* within the hypothalamic paraventricular nucleus of *Mrap2^tm1a/tm1a^* mice. Circulating levels of both high-density lipoprotein and low-density lipoprotein were significantly increased in *Mrap2* deficient mice. Taken together, these data corroborate the role of MRAP2 in metabolic regulation and indicate that, at least in part, this may be due to defective central melanocortin signalling.

## Introduction

Melanocortin receptor accessory protein (MRAP) and its paralogue MRAP2 are a recently identified class of small, single-pass transmembrane domain accessory proteins (Chan *et al*. 2009, Novoselova *et al*. 2013). Both MRAP and MRAP2 have been shown to interact with the melanocortin receptors (MCRs), a family of G protein-coupled receptors (GPCRs) with diverse physiological function stimulated by pro-opiomelanocortin (POMC) derived peptide agonists such as adrenocorticotropin hormone (ACTH) and α-MSH (Cone 2005, Chan *et al*. 2009). Of the five MCRs (MC1R–MC5R), only the function of the melanocortin 2 receptor (MC2R) is clearly recognized to be facilitated by MRAPs (Metherell *et al*. 2005, Chan *et al*. 2009), although *in-vitro* data suggests a broader role in conjunction with all the MCRs (Chan *et al*. 2009, Sebag & Hinkle 2009, 2010).

MRAP is highly expressed in the adrenal gland and is essential for MC2R function. Mutations in *MRAP* are associated with familial glucocorticoid deficiency (OMIM#607398) (Metherell *et al*. 2005). *MRAP2* is predominantly expressed in the central nervous system and hypothalamus, in particular within the paraventricular nucleus (PVN), a region known to have a role in energy homeostasis (Chan *et al*. 2009). Mice with global and brain-specific *Mrap2* deletion developed marked obesity and rare loss-of-function or missense heterozygous variants in *MRAP2* were also identified in humans with severe early-onset obesity (Asai *et al*. 2013). This work demonstrated that MRAP2’s role in the control of body composition and growth is via MC4R signalling (Asai *et al*. 2013). Further evidence for a link with MC4R signalling came from a study on the role of Mrap2 in zebrafish feeding and growth (Sebag *et al*. 2013).

Given these data, the phenotype observed in *Mrap2*-deficient mice is likely, at least in part, to be driven by disruption of central melanocortin signalling. However, some areas of uncertainty remain. In particular, the paradoxical observation that the mutant mice become obese without detectable changes in food intake or energy balance (Asai *et al*. 2013) requires exploration, as does the potential role of MRAP2 in peripheral cholesterol and lipid metabolism, a function known to be regulated by melanocortins (Nogueiras *et al*. 2007, Perez-Tilve *et al*. 2010). In this study, we have used an independently derived line of *Mrap2*-deficient mice (*Mrap2^tm1a/tm1a^*) on two different genetic backgrounds to further study the role of MRAP2 in the regulation of energy homeostasis and the control of cholesterol and lipid metabolism.

## Materials and methods

### Generation of *Mrap2*-deficient mouse

**Figure 1:**
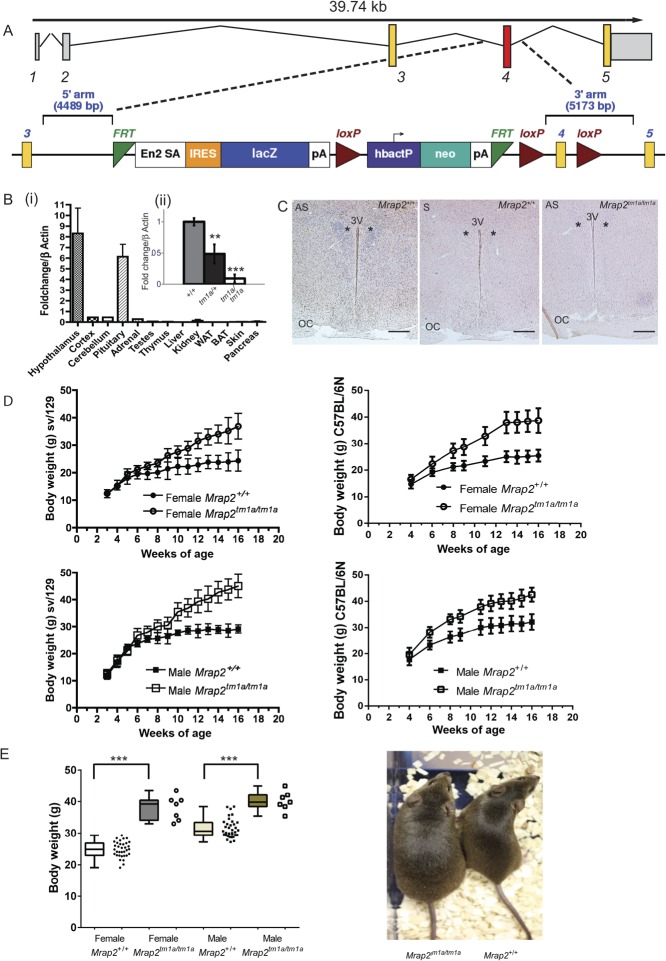
*Mrap2* gene disruption results in weight gain (A) Schematic of knockout-first strategy for *Mrap2*. A promoter driven cassette including lacZ and neo genes are inserted upstream of critical exon 4. (B) Q-RT-PCR in 129/Sv mice demonstrating (i) *Mrap2* expression in a range of wild-type mouse tissues showing the highest expression levels in the hypothalamus, *n*=3. (ii) reduced hypothalamic *Mrap2* transcript in *Mrap2^tm1a/+^*and *Mrap2^tm1a/tm1a^* compared with *Mrap2*^+/+^ mice (*n*=3 per genotype); mean plotted ± s.e.m.; **P<0.005; ***P<0.0005). (C) Expression of *Mrap2* in the hypothalamus of the wild-type 129/Sv *Mrap2*^+/+^ and *Mrap2^tm1a/tm1a^* mice as shown by *in situ* hybridization using coronal brain sections (approx. bregma – 0.6mm). AS, antisense probe, S, sense probe as a negative control. Third ventricle indicated as 3 V, asterisk indicates position of the PVN, OC, optic chiasm; scale bars=200μm. (D) Weight curves of *Mrap2^tm1a/tm1a^* in both genders and genetic backgrounds illustrated. 129/Sv *Mrap2^tm1a/tm1a^* *n* > 8 per genotype and gender, C57BL/6N *Mrap^2tm1a/tm1a^* *n*=7 of each gender and genotype. (E) Total body weight gain of C57BL/6N *Mrap2*^tm1a/tm1a^ mice by the age of 16weeks, *n*=7 for each gender/genotype (left) and appearance of the mutant mice compared with the wild type (right). Data is presented as both box-and-whiskers plot (showing min-mean-max values, with the box representing the 25th and 75th percentiles), and as a scatter dot plot for individual values. *P*-values presented on graphs are either global *P*-values for genotype adjusted for multiple correction testing, or (in the cases of sexual dimorphism) the *P*-value is the impact of genotype for that sex.

Mice carrying the knockout-first conditional-ready allele *Mrap2^tm1a(EUCOMM)Wtsi^* (abbreviated to *Mrap2^tm1a^*) were generated on a C57BL/6N background as part of the Sanger Mouse Genetics Project (MGP) (Fig. 1A). Mice carrying the same *Mrap2^tm1a^* allele were generated separately on a 129S5/SvEvBrdWtsi;129P2/OlaHsdWtsi background (abbreviated to 129/Sv). Detailed description of the Sanger Mouse Genetics Project methodology has been reported (Skarnes *et al*. 2011). Briefly, a promoter-containing cassette (L1L2_Bact_P) was introduced upstream of the critical *Mrap2* exon 4 at position 87175333 of Chromosome 9, Build GRCm38 (Fig. 1A). The vectors containing *Mrap2^tm1a^* were electroporated into C57BL/6N derived JM8F6 and 129P2/OlaHsd derived E14Tg2a embryonic stem (ES) cells. Correct ES cell gene targeting was confirmed by long-range PCR and quantitative PCR. Targeted ES cells were microinjected into blastocysts and used to generate chimeras. Germ-line transmission was confirmed by genotyping PCR analyses (http://www.knockoutmouse.org/kb/25/). Mice obtained from heterozygous intercross were genotyped for the *Mrap2^tm1a^* allele by PCR (Supplementary Table 1, see section on supplementary data given at the end of this article).

### Animal husbandry

The care and use of all animals were carried out in accordance with the UK Home Office regulations, UK Animals (Scientific Procedures) Act 1986. Mice were kept under a standard 12h light:12h darkness cycle with food and water *ad libitum* unless otherwise stated. 129/Sv background mice were maintained in a facility at 22°C and fed a standard chow (SDS RM3, Essex, UK). Mice on a C57BL/6N background were maintained at 21°C±2°C, humidity 55%±10% and fed a standard rodent chow (LabDiets 5021-3, IPS, Richmond, VA, USA).

### Metabolic phenotyping

Metabolic phenotyping was undertaken at two independent centres. In accordance to the 3R (replacement, reduction and refinement) principles of humane experimental technique and based on scientific objectives, not all procedures were performed on both lines. The genetic background of *Mrap2^tm1a^* mice used in each experiment have been specified previously.

Phenotyping using C57BL/6N mice was performed at the Wellcome Trust Sanger Institute as part of the MGP (White *et al*. 2013), whilst studies using 129/Sv background mice were carried out at the University of Cambridge Metabolic Research Laboratories (MRL). For data arising from the MGP, a cumulative baseline was generated from controls of the same genetic background, age and sex. Seven male and seven female *Mrap2^tm1a^* mice were processed in five batches for each sex (one to three mice per batch) and were phenotyped unblinded as part of a larger mixed genotype group that included weekly wild-type controls, with the individual mouse as the experimental unit. Animals for testing were randomly assigned to test sessions and operators. Mice were group housed to a minimum density of three per cage.

Body composition of 14-week-old anaesthetized C57BL/6N *Mrap2^tm1a^* mice were determined by dual-energy X-ray absorptiometry (DEXA) using a Lunar PIXImus2 mouse densitometer (General Electric Medical Systems, Fitchburg, WI, USA).

After overnight fasting (approximately 16h), intraperitoneal glucose tolerance tests (IPGTT) were carried out on 13-week-old mice. After taking a baseline glucose measurement, mice were given a single glucose injection (2g/kg) and blood glucose measured at 15, 30, 60 and 120min (Accu-Chek Aviva, Roche).

Blood for plasma biochemistry was collected from 16-week-old C57BL/6N animals into lithium-heparin tubes. Animals were not fasted unless otherwise indicated. Clinical blood chemistry was carried out using an Olympus AU400 chemistry analyser (Olympus). Insulin levels were measured by Mesoscale Discovery array technology platform.

Additional data relating to the C57BL/6N *Mrap2^tm1a/tm1a^* line can be found at http://www.mousepheno­type.org/data/genes/MGI:3609239. For studies at MRL, individual experiments were matched for age and sex of mice. The body weight and length were measured weekly since weaning. Food intake was carried out on 8-week-old single-housed acclimatized animals. Response to fasting was measured after mice were moved into clean cages, and food was removed at 07:00 for 24h.

Energy expenditure was determined at 8 weeks of age using indirect calorimetry. Animals were placed in a custom-built monitoring system based on their home cages (Ideas Studio, Cambridge, UK). Oxygen consumption and carbon dioxide production was measured, and samples were taken at 18min intervals for 48h. Energy expenditure was calculated using indirect calori­metry with the Elia and Livesey constants for respiratory quotie­nt (Elia & Livesey 1992). Activity was assessed by beam breaks (beams 1.25cm apart) and measurements were taken as total, rather than consecutive beam breaks.

### Behavioural tests

Open field assessment was used to quantify spontaneous locomotor behaviour in a novel environment. The open field, custom-designed-walled, infra-red backlit arena 75cm^2^ (Tracksys Ltd., Nottingham, UK) was subdivided into a centre zone (42 cm^2^) with the remainder designated as border zone. Twenty-week-old 129/Sv background mice were recorded for a 20min period using Noldus Ethovision-3 video tracking software. The position of the centre-point of the mouse within the open field was recorded. A mouse was considered to begin moving when its velocity surpassed 2 cm/s and stop moving when below 1.75 cm/s. *Mrap2^tm1a/tm1a^* and their *Mrap2^+/+^* controls were littermates housed in single-sex groups of three to five.

### Histology, non-radioactive *in-situ* hybridization, immunohistochemistry and PVN stereotaxic counts

For haematoxilin-eosin (H&E) staining, tissues were fixed in 4% paraformaldehyde (PFA) (Sigma), washed, dehydrated and embedded into paraffin before sectioning to 7µm. For Oil-Red-O staining, flash frozen liver was embedded into OCT (VWR), 10µm cryosections were adhered onto slides (Thermo Fisher) and stained with Oil-Red-O (Sigma). Both staining techniques were performed according to standard protocols. *Ucp1* immunohistochemistry was carried out using brown fat paraffin sections anti-*Ucp1* antibody (1/500) according to the manufacturer’s instructions (ab10983, Abcam) followed by detection using anti-rabbit HRP antibody (Thermo-Fisher) with DAB staining (Vector).

To generate riboprobes for *in-situ* hybridization (ISH), RNA was extracted from hypothalamus and cDNA prepared. Full-length *Mrap2* cDNA fragment (898 bp) was PCR amplified (Supplementary Table 1), ligated into pGEM-T easy vector (Promega), sequenced and then linearized with EcoRI or NotI (Promega). Digoxigenin (DIG)-labelled antisense and sense cRNA probes were synthesized by *in-vitro* transcription with T7 or SP6 RNA polymerases (Roche). Dissected brains were embedded into OCT and frozen in liquid nitrogen, 20µm cryosections were cut onto slides and fixed with ice-cold 4% PFA for 20min. Slides were then subjected to ISH as described previously (Gorrigan *et al*. 2011).

To determine the PVN neuron counts, brains of *Mrap2^tm1a/tm1a^* and wild-type littermates (three brains per group) were fixed in 4% PFA, cryoprotected with 20% sucrose and cryosectioned 20μm each, starting from −0.58mm to −1.22mm to bregma (Franklin & Paxinos 2012). After Nissl staining, the slides were visualized and images taken using Zeiss Axio Scope A1. The neurons within the PVN were then counted using ImageJ software (http://imagej.nih.gov/ij/).

### Laser-captured microdissection and RNA isolation

Mouse brains from 9-week-old, 129/Sv *Mrap2^tm1a/tm1a^* and *Mrap2^+/+^* mice were dissected, immediately embedded into OCT and frozen in liquid nitrogen. Coronal sections (20μm) covering the region from −0.58mm to −1.22mm caudal to bregma (Franklin & Paxinos 2012) were cut on a cryostat and mounted on Superfrost Plus slides (Thermo-Fisher). Frozen sections were fixed for 40s in 95% ethanol and then rehydrated (75% and 50% ethanol, 30s each). The slides were stained with 1% cresyl violet in 75% ethanol (w/v) for 45s, dehydrated in a graded ethanol series (50, 75, 95, 100% for 30s each), in 100% ethanol for 5min and air-dried. Laser microdissection was performed using a P.A.L.M. MicroBeam (Zeiss). The PVN was collected into AdhesiveCap tubes (Zeiss). Total RNA was immediately isolated using the RNAqueous-Micro Kit (Ambion). Quality and quantity of the total RNA samples were determined by the Agilent BioAnalyzer using PicoChip. RNAse free technique and RNAase free reagents were used throughout.

### RNA microarray hybridization and analysis

Fifteen nanograms of isolated RNA with the RNA Integrity Number of at least 6.5 (*n*=4 for *Mrap2*^+/+^; *n*=3 for *Mrap2^tm1a/tm1a^*) was converted into cDNA using Ovation PICO SL System V2 (NuGEN), which was then fragmented and labelled using Encore BiotinIL Module (NuGEN). About 1500ng of each labelled product was then hybridized with MouseRef-8v2.0 Expression BeadChip Kit according to the manual and scanned using iScan (Illumina, San Diego, CA, USA). Raw image data were converted to *bsc* format using Illumina GenomeStudio 2011.1 software. Bonferroni correction with Family-Wise Error Rate (FWER) of 0.05 was applied to identify statistical significance of gene expression changes. Pathway analysis was performed using DAVID6.7 (http://david.abcc.ncifcrf.gov/tools.jsp) and STRING 10 (http://string-db.org/).

### Quantification of RNA by real-time quantitative PCR (Q-RT-PCR)

Dissected tissues were immediately frozen in liquid nitrogen, homogenized using Precellys24 (Precellys, Bertin Technologies, Paris, France) into RPL buffer (Qiagen) and the RNA was extracted with RNeasy Mini Kit (Qiagen). cDNA was produced with SuperScriptII (Life Technologies) and 50ng of cDNA was used for RT-Q-PCR with TaqMan Universal MasterMix II and gene-specific TaqMan probes (Life technologies, Supplementary Table 2). The fold change related to Actin-b was calculated using 2^-^^ΔΔTh^ method (Livak & Schmittgen 2001).

### Protein quantification

White and brown fat tissues were homogenized using Precellys24 in ice-cold RIPA buffer (Sigma) containing phosphotase (Roche) and protease inhibitors cocktail (Sigma). Lysates were centrifuged for 20min at 4°C before separation of the lipid layer. The SDS–PAGE samples were prepared with 2× Sample buffer (Sigma), heated at 95°C for 5min, centrifuged for 20min at 4°C to separate samples from residual lipids and subjected to western blotting. The membrane was blocked with 5% bovine serum albumin in TBS (Life Technologies) for 1h at 22°C followed by incubation at 4°C overnight with the primary antibody: anti-ACTB antibody 1/10,000 (Abcam), anti-UCP1 1/5000 (Abcam) and antibodies for Fatty Acid and Lipid Metabolism and Lipolysis Activation (8334, 8335 Cell Signaling Technology). After three washes, the membranes were probed with anti-mouse 680 and anti-rabbit 800 IRDye antibodies (LI-COR). The band intensities were quantified using Odyssey software.

### Statistical analysis

All data were generated from the MGP utilized statistical analysis with RStudio running R version 3.1.2 and Phenstat package version 2.0.1. This uses a mixed-model framework (Karp *et al*. 2012) to assess the impact of genotype on phenotype. The analysis was performed by loading the model without body weight, therefore analysing the absolute differences between genotypes whilst accounting for sex, using the model: Y=Genotype+Sex+Genotype×Sex. Multiple correction testing was performed on the global *P*-values using the Hochberg correction. Data is presented as both box-and-whiskers plot (showing min-mean-max values, with the box representing the 25th and 75th percentiles), and as a scatter dot plot for individual values. *P*-values presented on graphs are either global *P*-values for genotype adjusted for multiple correction testing, or (in the cases of sexual dimorphism) the *P*-value is the impact of genotype for that sex.

For other data, males and females were assessed independently and the effect of genotype compared with wild-type controls was statistically tested using a two-tailed Student’s *t*-test. For calorimetry data, multiple linear regression analysis (ANCOVA) was used. Data is plotted as mean±s.e.m. and analysed using Microsoft excel and GraphPad Prism.

## Results

### Production of *Mrap2*-deficient mice

Mice carrying the mutant *Mrap2^tm1a^* allele were viable with expected homozygous mutant offspring born from heterozygous matings (21% C57BL/6N *Mrap2^tm1a/tm1a^* and 23% 129/Sv *Mrap2^tm1a/tm1a^*). Both female and male *Mrap2^tm1a/tm1a^* mice were fertile and did not exhibit any changes in skin or hair colour/appearance. The introduction of the knockout-first *Mrap2^tm1a^* allele resulted in targeted disruption of the critical exon 4 encoding the transmem­brane domain of the protein. The predicted outcome would be a premature stop codon, thus producing a short 132bp transcript that, if translated, would produce a 44 amino acid protein (predicted MW 5 kDa). Previous work demonstrated that such a protein was unlikely to be translated (Asai *et al*. 2013). However, generation of hypomorphic mice have been demonstrated previously using the ‘knockout-first’ strategy targeting other genes (McIntyre *et al*. 2012, Chen *et al*. 2013, White *et al*. 2013). We therefore determined the expression of *Mrap2* by Q-RT-PCR analysis using a TaqMan probe spanning exons 4–5. cDNA generated from whole hypothalamus extracted from mice on an 129/Sv background revealed a low but detectable residual *Mrap2* transcript of *Mrap2^tm1a/tm1a^* within homozygous mice (13%, range 11–16%), whilst heterozygotes *Mrap2^tm1a/+^* mice had approximately half of the *Mrap2* transcript expression compared with *Mrap2^+/+^* (Fig. 1B).

### *Mrap2* is predominantly expressed in the paraventricular nucleus of the hypothalamus

*Mrap2* RNA expression was studied in wild-type mice tissues (Fig. 1B). The highest expression level was detected in the hypothalamus with substantial expression observed in the pituitary gland. *Mrap2* expression was also detected in the cortex, cerebellum and adrenal gland. Kidney, testes, thymus and pancreas had very low expression levels whilst expression in white fat, liver, brown fat and skin was undetectable (Fig. 1B). ISH using a full-length *Mrap2* probe showed visible *Mrap2* RNA expression in the PVN of *Mrap2^+/+^* mice on a 129/Sv background, which was absent in *Mrap2^tm1a/tm1a^* mice (Fig. 1C).

### *Mrap2* deficiency results in obesity in both C57BL/6N and 129/Sv background

Before weaning, there was no difference in body weight between wild-type and *Mrap2^tm1a/tm1a^*mice (Fig. 1D). However, in both genetic backgrounds and in both sexes, *Mrap2^tm1a/tm1a^* mice had a significant increase in body weight from 6 weeks of age. By 16 weeks of age on a C57BL/6N background, mean body weight in male wild-type mice was 32.2g, compared with 42.5g in *Mrap2^tm1a/tm1a^* mice; the corresponding weights in females were 25.4g and 38.7g, respectively (Fig. 1E). Similarly, on a 129/Sv background, mean body weight in male wild-type mice was 29.1g, compared with 45.1g in *Mrap2^tm1a/tm1a^* mice; the corresponding weights in females were 24.4g and 36.9g, respectively.

**Figure 2:**
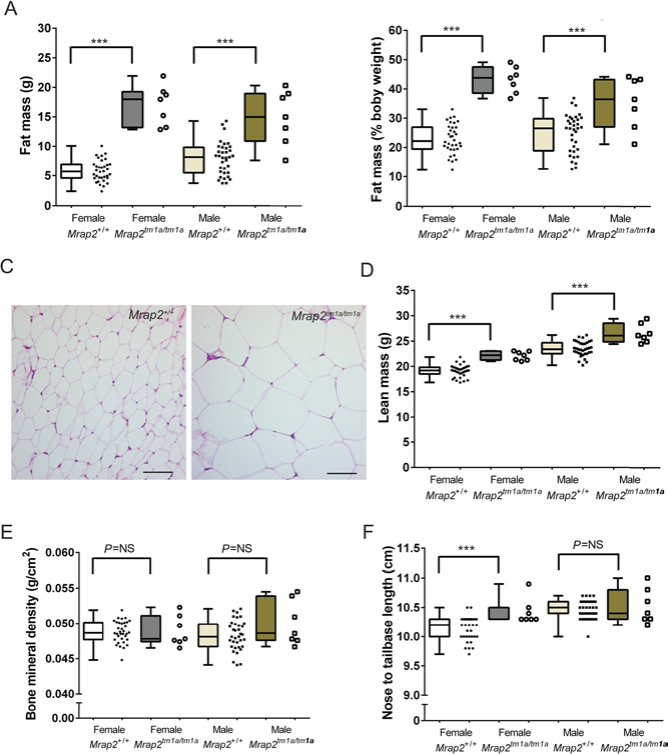
Increased fat content and lean mass in *Mrap2^tm1a/tm1a^* mice at 14 weeks. (A) Increased fat mass in mutant C57BL/6N mice. (B) Fat mass represented as a percentage of body weight. (C) Adipocyte hypertrophy as demonstrated by H&E histology of peripheral fat (representative image of inguinal white fat from 129/Sv mice). Scale bars=200μm. (D) Increased lean mass and (E) no difference in bone mineral density or content (data not shown) in *Mrap2^tm1a/tm1a^* mice (C57BL/6N) compared with *Mrap2^+/+^*. (F) Increased body length in the female mutant mice only (C57BL/6N). *n*=7 for each *Mrap2^tm1a/tm1a^* group, 34 for female *Mrap2^+/+^* and 35 for *Mrap2^+/+^* controls gender/genotype. *P*-values presented on graphs are either global *P*-values for genotype adjusted for multiple correction testing, or (in the cases of sexual dimorphism) the *P*-value is the impact of genotype for that sex. ***P=0.0005; NS, not significant.

In C57BL/6N, this increase in body weight was as a result of a significant increase in both fat and lean mass (Fig. 2A and D). C57BL/6N *Mrap2^tm1a/tm1a^* females had twice the fat/body weight ratio of *Mrap2^+/+^* controls and *Mrap2^tm1a/tm1a^* males displayed a 1.5-fold increase (Fig. 2A and B) with clear increase in adipocyte size macroscopically (Fig. 2C). No difference in bone mineral content or density was observed in either sex (Fig. 2E). Body length was signi­ficantly increased in female, but not male *Mrap2^tm1a/tm1a^* mice (Fig. 2F). On the 129/Sv background, a significant increase in fat mass was recorded in both male and female *Mrap2^tm1a/tm1a^* mice compared with wild-type controls with no change in lean mass observed (data not shown).

### *Mrap2*-deficient mice display little difference in food intake and energy expenditure compared with wild-type control mice

**Figure 3:**
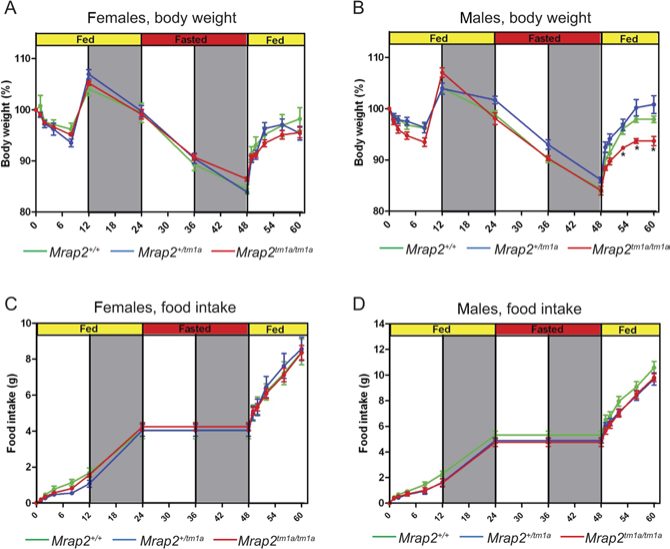
Food intake and energy expenditure balance in *Mrap2^+/+^*, *Mrap2^tm1a/+^* and *Mrap2^tm1a/tm1a^* mice. Body weight dynamics of female (A) *Mrap2*^+/+^ *n*=6; *Mrap2^tm1a/+^* *n*=4; *Mrap2^tm1a/tm1a^* *n*=10 and male (B) *Mrap2^+/+^* *n*=6; *Mrap2*^tm1a/+^ *n*=7; *Mrap2^tm1a/tm1a^* *n*=6 mutant mice and their food intake (C and D) in response to a 24h fast (129/Sv background). *P*<0.05.

Activation of the melanocortin system has a role in both feeding behaviour in both *ad libitum* conditions and in re-feeding after fasting. To determine if loss of *Mrap2* affects feeding behaviour in either situation, the food intake and body weight of 8-week-old male and female 129/Sv mice were monitored over a period of 60h. For the first 24h, food was freely available, after which mice were fasted for 24h followed by reintroduction of food (Fig. 3A, B, C and D).

Neither female nor male *Mrap2^tm1a/tm1a^* mice exhibited changes in food intake when compared with wild-type controls over the entire period. Further, *Mrap2^tm1a/tm1a^* mice did not show any difference in the rate of weight loss upon fasting compared with the wild-type mice. However, interestingly, during re-feeding after a fast, *Mrap2^tm1a/tm1a^* male mice did not re-gain weight as fast as the wild-type males (Fig. 3C and D).

Total energy expenditure measurements versus lean mass or total body weight did not show significant changes between the genotypes/sex (Supplementary Fig. 1). Analysis of respiratory quotient over a period of 48-h demonstrated that it did not differ between *Mrap2^tm1a/tm1a^* and their *Mrap2^+/+^* littermates (Supplementary Fig. 2). In keeping with a lack of change in energy expenditure, there was no difference between *Mrap2^tm1a/tm1a^* and *Mrap2^+/+^* mice in the expression level of *Ucp1* mRNA and UCP1 protein levels in brown adipose tissue of age-matched animals, despite differences in morphology (Supplementary Fig. 3).

**Figure 4:**
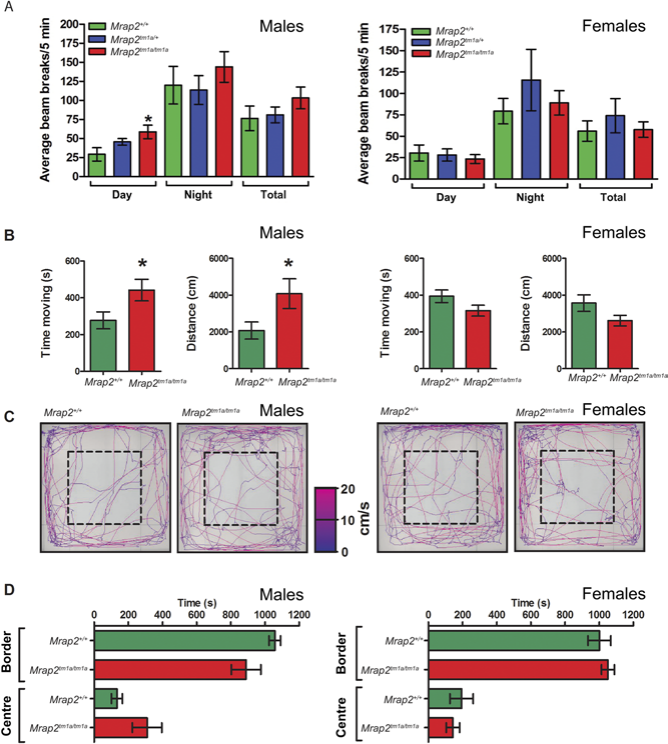
Male *Mrap2^tm1a/tm1a^* mice locomotor activity analysis. (A) Beam breaks/5 min in male (left, *Mrap2^+/+^* *n*=9; *Mrap2^tm1a/+^* *n*=7; *Mrap2^tm1a/tm1a^* *n*=11) and female (right, *Mrap2*^+/+^ *n*=6; *Mrap2^tm1a/+^* *n*=4; *Mrap2^tm1a/tm1a^* *n*=10) mice are shown, with male *Mrap2^tm1a/tm1a^* mice demonstrating significantly increased locomotor activity in their home cages during the daytime. (B) Open field assessment of *Mrap2^tm1a/tm1a^* mice also indicate a significant increase in total time moving and distance travelled over 20 min in males (left), when compared with *Mrap2*^+/+^ littermates, but not females (right). (C) Representative activity traces of the centre-point of individual male mice (left) and female mice (right) in the open field. The colour of the trace indicates the velocity of the mouse from 0cm/s (blue) to 20cm/s (pink). The centre of the open field is indicated by a dashed box. (D) Neither male (left) nor female *Mrap2^tm1a/tm1a^* mice (right) displayed a significant difference in time spent in areas of the open field, compared with wild-type controls. *N* number for B–D is eight per group/gender, *P<0.05.

Locomotor activity measurements (average beam breaks in a 5min time period) demonstrated that male 129/Sv *Mrap2^tm1a/tm1a^* mice, compared with wild-type, moved significantly more during the daytime (Fig. 4A). No difference was observed in females.

### *Mrap2*^tm1a/tm1a^ mice display behavioural changes when presented with a novel environment

To further examine the locomotor activity as well as novel environment exploration and anxiety-related behaviour, 20-week-old 129/Sv *Mrap2^tm1a/tm1a^* mice were subjected to an open field exploration test during the light phase. This recapitulated the sex-specific difference in locomotor activity between female and male *Mrap2^tm1a/tm1a^* mice (Fig. 4B and C). *Mrap2^tm1a/tm1a^* male mice spent more time moving and covered a greater distance compared with *Mrap2^+/+^* mice. Although *Mrap2^tm1a/tm1a^* male mice appeared to spend more time traversing the centre of the open field than controls, the difference was not significant, *P*=0.075 (Fig. 4C and D). There was no difference in thigmotactic behaviour in the females, and *Mrap2^tm1a/tm1a^* mice of both sexes displayed no differences in gait, circling and rearing behaviour (data not shown).

### *Mrap2*-deficient mice are *Sim1* deficient

**Figure 5:**
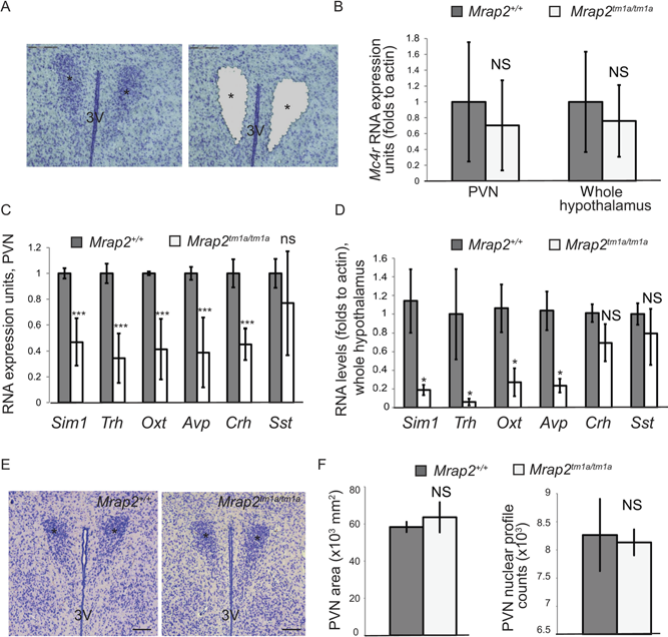
*Mrap2* is involved in *Mc4r* regulation in the hypothalamus. (A) An example of the hypothalamic section stained with cresyl violet before the microdissection (left panel) and after (right panel). Asterisks show the position of the PVN, 3 V-third ventricle, the scale bars are 150 μm. (B) *Mc4r* expression level in the PVN (*Mrap2*^+/+^ *n*=3, *Mrap2^tm1a/tm1a^* *n*=3) and in the whole hypothalamus (*Mrap2*^+/+^ *n*=4, *Mrap2^tm1a/tm1a^* *n*=4) as determined by the qPCR. (C) Expression of *Sim1*, *Trh*, *Oxt*, *Avp*, *Crh* and *Sst* in the PVN of 129/Sv wild type (*n*=4) and *Mrap2^tm1a/tm1a^* (*n*=3) mice. The data are represented as the mean of the microarray fluorescence values (±s.e.m.), normalized to the wild type for each gene. *P<0.05; **P<0.005; ***P<0.0005. (D) Expression of *Sim1*, *Trh*, *Oxt*, *Avp*, *Crh* and *Sst* in the whole hypothalamus of the wild-type and *Mrap2^tm1a/tm1a^* mice as determined by the qPCR. Data from male mice *n*=4 per genotype is shown. (E) Morphology of the PVN of 129/Sv *Mrap2^tm1a/tm1a^* mice (right panel) compared with the wild type as shown by representative images of coronal brain sections (approx. bregma –0.8 mm) stained by Nissl. (F) Average PVN area size (left graph) and stereotaxic counts of Nissl positive cells (right graph) in the PVN of the mutant 129/Sv mice (*n*=3) and their wild-type littermates (*n*=3).

To further explore what might be the driving changes in body composition in *Mrap2*-deficient mice, we undertook transcriptomic analysis of laser microdissection PVN from 9-week-old 129/Sv *Mrap2^tm1a/tm1a^* mice and wild-type littermates (Fig. 5A). Mice of a 129/Sv genetic background, less prone to developing obesity-related co-morbidity, were used to reveal the effect of *Mrap2* deficiency without secondary changes caused by hyperinsulinaemia and/or elevated glucose. We confirmed the changes observed in laser-capture material by undertaking Q-RT-PCR on whole hypothalamus extracted from a separate, second population of 129/Sv *Mrap2^tm1a/tm1a^* mice and wild-type littermates. Expression of genes that did not show any changes by microarray, such as *Sf-1* and *Pomc*, were also confirmed by Q-RT-PCR as additional controls (Supplementary Fig. 4).

We could not detect significant changes in *Mc4r* mRNA expression in the PVN of *Mrap2^tm1a/tm1a^* mice due to the high variability between mice within each group (Fig. 5B). However, we found that *Sim1* mRNA level in the PVN of *Mrap2^tm1a/tm1a^* mice was less than 50% of that observed in wild-type littermates (Fig. 5C). *Sim1* is responsible for the late stages of the differentiation of oxytocin (Oxt), arginine vasopressin (Avp), corticotrophin-releasing hormone (Crh), thyrotropin-releasing hormone (Trh) and somatostatin neurons (Sst) (Michaud *et al*. 1998). In keeping with this, in *Mrap2^tm1a/tm1a^* mice, PVN expression levels of *Ox*t, *Avp*, *Trh* and *Crh* were significantly decreased compared with the wild-type. *Sst* expression in *Mrap2^tm1a/tm1a^* was unchanged compared with *Mrap2^+/+^* mice, although results were variable within the cohort (*n*=3 per group). Analysis of RNA from whole hypothalami (Fig. 5D) recapitulated these findings, except for *Crh* expression levels, which did not reach statistical significance. All changes were confirmed in both sexes (data not shown).

It is known that SIM1 is implicated in the development of the PVN and *Sim1*^+/^^–^ mice exhibit a smaller PVN with reduced neuron number compared with their wild-type littermates (Michaud *et al*. 2001). We could not find morphological changes or a reduction in the number of neurons in the PVN of *Mrap2^tm1a/tm1a^* mice compared with wild-type (Fig. 5E and F), suggesting that unlike *Sim1*^+/^^–^, a lack of *Mrap2* does not cause underdevelopment of the PVN.

### *Mrap2* deficiency increases circulating HDL and LDL cholesterol

**Figure 6:**
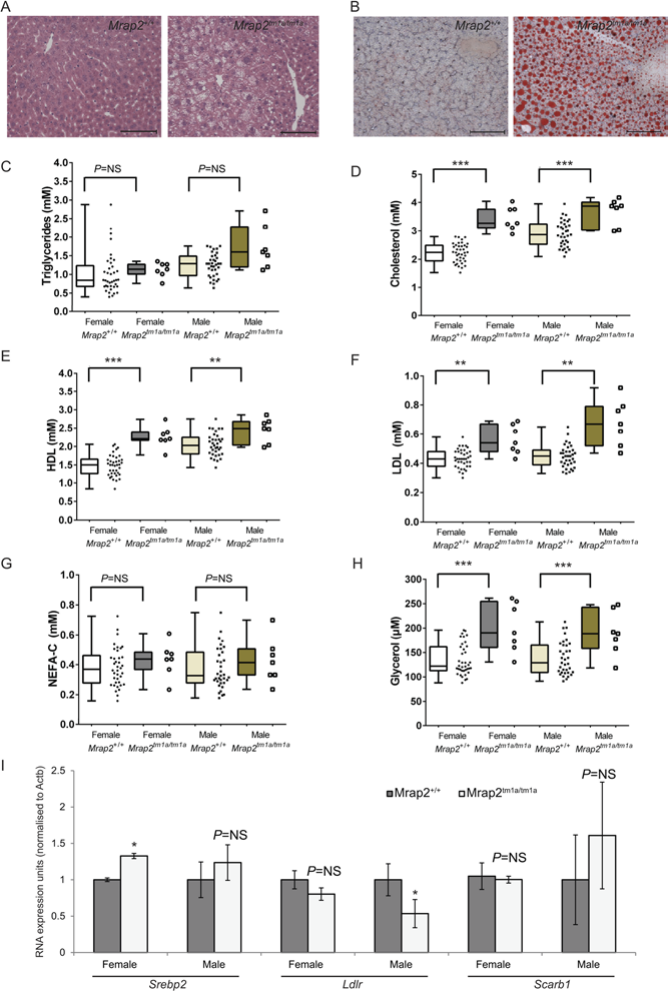
*Mrap2^tm1a/tm1a^* mice exhibit fatty liver and changes in cholesterol metabolism. Morphological changes in the liver of 129/Sv *Mrap2^tm1a/tm1a^* mutant mice as shown by H&E staining (A) and Oil Red O (B), suggesting lipid accumulation in *Mrap2^tm1a/tm1a^*. Scale bars=200μm. (C, D, E, F, G and H) Circulating TAG, total cholesterol, HDL, LDL, NEFA-C and Glycerol in 16-week C57BL/6N *Mrap2^tm1a/tm1a^* mice are shown, *n*=7 for each *Mrap2^tm1a/tm1a^* group, 38 for female *Mrap2^+/+^* and 35 for *Mrap2^+/+^* controls gender/genotype. The *P*-values presented on graphs are either global *P*-values for genotype adjusted for multiple correction testing, or (in the cases of sexual dimorphism) the *P*-value is the impact of genotype for that sex. NS, not significant. *P<0.05; **P<0.005; ***P<0.0005; NS, not significant. (I) Elevated expression levels of *Srebp2* in *Mrap2^tm1a/tm1a^* female mice, reduced *Ldlr* in male *Mrap2^tm1a/tm1a^* mice and expression levels of *Scarb1* in female mice and male mice (*n*=4 for each genotype/gender, *P<0.05, NS, not significant).

Macroscopically, the livers of *Mrap2^tm1a/tm1a^* mice were visibly pale in both sexes in both 129/Sv and C57BL/6N backgrounds, and the histological analysis showed lipid accumulation (Fig. 6A and B). There is recent evidence that the central melanocortin system directly controls peripheral lipid metabolism and circulating cholesterol (Nogueiras *et al*. 2007, Perez-Tilve *et al*. 2010). We therefore studied the cholesterol and lipid profile in C57BL/6N *Mrap2^tm1a/tm1a^* mice. The blood triacylglyceride levels (TAG) were not significantly different in *Mrap2^tm1a/tm1a^* mice compared with wild-type (Fig. 6C). However, total circulating cholesterol in *Mrap2^tm1a/tm1a^* mice was significantly higher than in wild-type controls of both sexes (Fig. 6D). HDL was elevated in both sexes with a greater percentage increase in females (Fig. 6E). LDL was significantly increased in male and female *Mrap2^tm1a/tm1a^* mice (Fig. 6F). NEFA-C levels were not significantly different between *Mrap2^tm1a/tm1a^* and *Mrap2^+/+^* mice of either sex (Fig. 6G), whilst glycerol concentration was increased to a similar degree in mutant mice of both sexes (Fig. 6H). To investigate whether high cholesterol levels were due to a decrease in cholesterol re-uptake in the liver or an increase in cholesterol synthesis, we analysed the expression of the HDL scavenger receptor *Scarb1*, LDL receptor (*Ldlr*) and the key transcription factor of cholesterol biosynthesis *Srebp2* (Shimomura *et al*. 1998) in the livers of 129/Sv *Mrap2^tm1a/tm1a^* mice. Interestingly, *Srebp2* mRNA levels were increased in the livers of female *Mrap2^tm1a/tm1a^* mice, whereas *Scarb1* and *Ldlr* levels were similar to the wild type (Fig. 6I). Expression analysis of livers from male *Mrap2^tm1a/tm1a^* mice showed lower levels of *Ldlr* mRNA whereas *Scarb1* and *Srebp2* transcript were similar to the wild-type male littermates (Fig. 6I).

**Figure 7:**
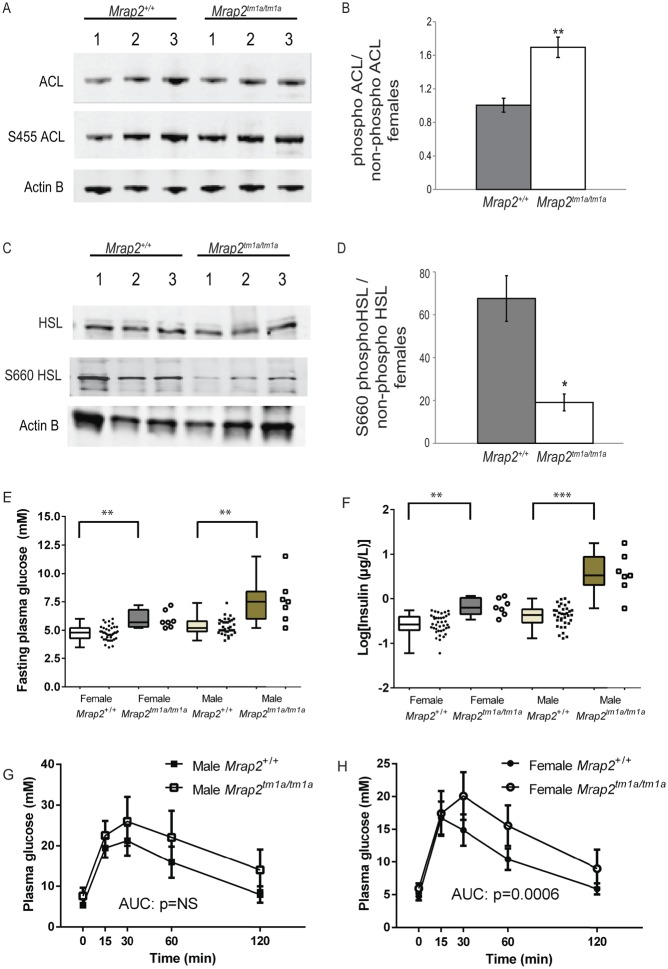
Phosphorylation of ACL and HSL are changed in female *Mrap2^tm1a/tm1a^* mice and insulin insensitivity in both genders observed at 13 weeks of age. (A) Analysis of ACL phosphorylation in white fat of the female mutant mice compared with the wild type by immunoblotting. (B) A significant increase is demonstrated in mutant mice by using band densitometry analysis of the ratio of phosphorylated to non-phosphorylated ACL normalized to β-actin. (C) Phosphorylation of HSL on S660 in white fat of the female mutant mice is decreased as assessed by western blotting. (D) Densitometry analysis of phosphorylated to non-phosphorylated HSL normalized to β-actin was calculated (*n*=3 per genotype; 129/Sv). (E) Fasting plasma glucose and (F) fed-state plasma insulin are higher in C57BL/6N *Mrap2^tm1a/tm1a^* mutant mice of both genders associated with significantly elevated insulin levels (log transformed due to the range of values observed in *Mrap2^tm1a/tm1a^* male mice. (G&H) IPGTT performed on 13-week-old mice of both genders, delayed glucose clearance demonstrated in *Mrap2^tm1a/tm1a^* female mice but not statistically significant in male *Mrap2^tm1a/tm1a^* mice. *n*=7 for each *Mrap2^tm1a/tm1a^* group, 39 for female *Mrap2^+/+^* and 35 for *Mrap2^+/+^* controls gender/genotype. *P*-values presented on graphs are either global *P*-values for genotype adjusted for multiple correction testing, or (in the cases of sexual dimorphism) the *P*-value is the impact of genotype for that sex. AUC, area under the curve; *P<0.05; **P<0.005; ***P<0.0005; NS, not significant.

To study white fat function, we tested the protein levels and phosphorylarion state of enzymes involved in lipogenesis and fatty acid synthesis as well as phosphorylation of the rate-limiting enzyme for lipolysis of hormone sensitive lipase (HSL). Phosphorylation of ATP-citrate lyase (ACL), an enzyme responsible for the synthesis of cytosolic acetyl-CoA that serves lipogenesis and cholesterolgenesis pathways (reviewed in Chypre *et al*. 2012), was increased in white fat of female *Mrap2^tm1a/tm1a^* mice but not in *Mrap2^tm1a/tm1a^* male mice (Fig. 7A and B, Supplementary Fig. 5A and B).

HSL phosphorylation is known to be important for the enzyme activation and therefore lipolysis (Egan *et al*. 1992). The proportion of HSL phosphorylated on S563, S565 and S660 was analysed and it was found that the proportion of S660 phosphorylated HSL was three times lower in white fat of female *Mrap2^tm1a/tm1a^* mice compared with the wild type (Fig. 7C and D). Phosphorylation on other residues was not changed and male *Mrap2^tm1a/tm1a^* mice did not have changes in HSL phosphorylation on any residues tested (Supplementary Fig. 5C, D, E and F).

### Analysis of glucose homeostasis in *Mrap2*-deficient mice

*Mrap2^tm1a/tm1a^* mice of both sexes on a C57BL/6N background fed on a chow diet from weaning display elevated fasting plasma insulin concentration with higher fasting blood glucose at 13 weeks of age compared with *Mrap2^+/+^* mice (Fig. 7E and F). Glucose clearance in response to an IP glucose bolus (Fig. 7G and H) appeared delayed, although statistical analysis of the area under the curve was only significant in female mice.

## Discussion

In this study, we report the generation and characterization of a murine model with a targeted *Mrap2* allele (knockout-first *Mrap2^tm1a(EUCOMM)Wtsi^*). The construct used here is a *Tm1a* allele, which theoretically can still allow transcriptional read through (White *et al*. 2013). We detected low *Mrap2* expression within *Mrap2^tm1a/tm1a^* homozygous mice and in the absence of a suitable antibody we cannot rule out the possibility that the animals studied were indeed ‘strong hypomorphs’ rather than of complete knockouts.

*Mrap2^tm1a/tm1a^* mutant mice on both C57/BL6N and 129/Sv background display severe early-onset obesity with a significantly increased fat mass, consistent with a recent report of *Mrap2*-knockout mice (Asai *et al*. 2013). Unlike *Mrap2*^–^*^/^*^–^ mice on a 129/Sv genetic background (Asai *et al*. 2013), our C57BL/6N *Mrap2^tm1a/tm1a^* display elevated fasting insulin and blood glucose concentrations. We believe that this is an evidence of an interesting interaction between the genetic backgrounds with *Mrap2*, which will form the basis of a future study.

*Mrap2*^–^*^/^*^–^ mice have been reported previously to show no increased food intake or reduction in energy expenditure and thermogenesis to account for their increased body weight. In our assessment of 8-week-old *Mrap2^tm1a/tm1a^* mice, we recapitulated and confirmed these findings. We calculate that female *Mrap2^tm1a/tm1a^* mice gained more weight compared with the wild-type mice (females: 0.117±0.041g/day; males: 0.096±0.023g/day). To achieve this, a female mutant mouse would need to deposit 0.701±0.246kcal/day and a male mouse 0.578±0.139kcal/day (Flatt 1991). This would equate to an increase in food intake of 0.232±0.081g of standard chow (for females) and 0.191±0.046g (for males), which is within the measurable limits of food intake variation. Therefore, it is possible that the causative difference is below the threshold of the detection (Tschop *et al*. 2012, Speakman 2013). Indeed, older animals that are significantly more obese than their wild-type counterparts, and as a result would be expected to consume larger quantities, demonstrate a subtle increase in cumulative food intake when monitored over 50 days (Asai *et al*. 2013). Additionally, our behavioural tests on separate cohorts of animals, independently analysed on two separate platforms, demonstrated sex-specific increased daytime locomotor and exploratory activity in *Mrap2^tm1a/tm1a^* male mice, which may indicate food-seeking behaviour. These lines of evidence would point to hyperphagia being the key driver in the development of obesity. However, importantly, obesity in *Mrap2-*deficient animals clearly precede any change in food intake and in paired feeding studies *Mrap2-*deficient animals continue to gain more weight than their wild-type counterparts (Asai *et al*. 2013). It is only when further food restriction was undertaken did weight gain in mutant mice become equivalent to that of wild-type mice (Asai *et al*. 2013). Intriguingly, this suggests a far more complex mechanism at play in *Mrap2* null mice.

Our transcriptomic analysis of the PVN of the mutant mice also favoured increased energy intake as being a more likely promoter of increased body weight. It was found that *Sim1* expression levels were low in the PVN of the *Mrap2^tm1a/tm1a^* mice resembling *Sim1* deficiency. The reduced expression of *Sim1* and its associated neuropeptides cannot be secondary to obesity alone, as this was not observed in reported hypothalamic microarray data from obese mice fed in a high fat diet (Lee *et al*. 2010). SIM1 is a transcription factor that regulates development of the PVN, and *Sim1*^–^*^/^*^–^ mice die due to the abnormal hypothalamic architecture (Michaud *et al*. 2001). Heterozygous *Sim1^+/^*^–^ mice exhibit a small PVN with reduced neuronal number and develop severe early-onset obesity due to hyperphagia and increased linear growth. They have an impaired response to MTII, an MC3R/MC4R agonist, indicative of a disrupted central melanocortin pathway (Holder *et al*. 2004, Kublaoui et al. 2006a, b, Tolson *et al*. 2010). Expression analysis of the PVN from *Sim1*^+/^^–^ mice has shown an 80% decrease in *Oxt* expression and 20–40% decrease in *Trh, Crh, Avp* and *Sst* expression (Kublaoui *et al*. 2008). Compared with *Sim1^+/^*^–^ mice, we did not detect morphological changes within the PVN of *Mrap2^tm1a/tm1a^* mice. However, the levels of *Oxt*, *Avp* and *Trh* in *Mrap2^tm1a/tm1a^* mice PVN were reduced, consistent with low *Sim1* expression levels. Interestingly, despite these changes and also the high expression of *Mrap2* in the pituitary, we found no evidence of pituitary dysfunction in *Mrap2^tm1a/tm1a^* mice. Progression through puberty and fertility appear unchanged in mutant mice, and thyroid hormone levels, T3 and T4, were normal (data not shown). Corticosterone concentrations were previously reported to be normal (Asai *et al*. 2013). This would suggest that the level of neuropeptide expression is sufficient for peptide production and physiological stimulation of downstream hormones, as exemplified by normal levels of T4 in the case of *Trh*.

In contrast to the downstream effects, the change in neuropeptide expression is likely to play a direct role in maintaining energy homeostasis as it is known that *Oxt*, *Avp* and *Trh* in the PVN have anorexigenic effects (reviewed in Valassi *et al*. 2008), and *Oxt* is thought to be key to the mechanism for the hyperphagia of *Sim1^+/−^* mice (Kublaoui *et al*. 2008). Overall, the changes in *Sim1* provide further evidence that a central melanocortin pathway deficiency exists in *Mrap2^tm1a/tm1a^* mice as SIM1 has been considered to be downstream of MC4R signalling (Holder *et al*. 2004, Kublaoui *et al*. 2006*a*, *b*, Tolson *et al*. 2010).

Modulation of MC4R has been shown to directly affect peripheral lipid metabolism. *Mc4r*^–^*^/^*^–^ mice had elevated plasma cholesterol and HDL levels (Nogueiras *et al*. 2007, Perez-Tilve *et al*. 2010). Both sexes of *Mrap2^tm1a/tm1a^* mice displayed elevated circulating cholesterol although there is a suggestion that males and females partition cholesterol into HDL differently, consistent with reports of sex differences in the hepatic control of cholesterol metabolism (De Marinis *et al*. 2008). *Mrap2^tm1a/tm1a^* female mice showed increased *de novo* hepatic lipogenesis; however, unlike female *Mrap2^tm1a/tm1a^* mice, male *Mrap2^tm1a/tm1a^* mice had low expression of liver LDL receptor, possibly reflecting elevated circulating LDL levels.

*Mrap2^tm1a/tm1a^* female mice had increased ACL phosphorylation in white fat, a key modification that activates ACL catalytic activity (Berwick *et al*. 2002) and leads to an increase in *de novo* lipogenesis. Along with this change we found that phosphorylation of HSL on S660, which is phosphorylated by protein kinase A upon sympathetic nervous system activation (Anthonsen *et al*. 1998), was decreased. It is possible that both changes in the liver and white adipocytes are due to the low sympathetic tone which is in part regulated via the central melanocortin system (Nogueiras *et al*. 2007, Perez-Tilve *et al*. 2010). *Mrap2* is not expressed in white fat or liver and therefore is unlikely to influence *de novo* lipogenesis directly in these tissues, suggesting that MRAP2 may contribute to the melanocortin regulation of sympathetic outflow. The changes observed in white fat were only found in females and are thus unlikely to be the primary cause of MRAP2-associated obesity, although this might explain the greater severity of obesity in females.

Our study corroborates the role of MRAP2 in metabolism. The changes in cholesterol metabolism and transcriptomic profile in the PVN of *Mrap2^tm1a/tm1a^* mice support the notion that MRAP2 is involved in the MC4R signalling pathway *in vivo*. However, our data further highlights phenotypic differences between *Mrap2^-^*deficient and *Mc4r-*deficient mice. Despite both mice developing severe early-onset obesity, *Mc4r*^–^*^/^*^–^ mice are clearly hyperphagic with decreased energy expenditure (Huszar *et al*. 1997, Balthasar *et al*. 2005), whilst *Mrap2-*deficient mice display no demonstrable hyperphagia or reduction in energy expenditure. We describe other additional pheno­typic differences such as normal bone mineral content and density in *Mrap2^tm1a/tm1a^* mice in contrast to increased bone density in *Mc4r*^–^^/^^–^ mice (Braun *et al*. 2012). Importantly, Asai and coworkers demonstrated that *Mrap2*^–^*^/^*^–^ mice remain responsive to treatment with MTII, a MC3R/MC4R agonist, whilst the anorexic response to MTII is abolished in *Mc4r*^–^*^/^*^–^ mice, suggesting at least some preservation of MCR function centrally (Marsh *et al*. 1999, Asai *et al*. 2013). We also show sex-specific differences in glucose handling as well as an exploratory activity phenotype in *Mrap2^tm1a/tm1a^* mice. Taken together, our study points towards the likelihood of MC4R-independent mechanisms and possibly MCR-independent pathways in the pathogenesis of MRAP2-associated obesity.

## Supplementary data

This is linked to the online version of the paper at http://dx.doi.org/10.1530/JOE-16-0057.

## Declaration of interest

The authors declare that there is no conflict of interest that could be perceived as prejudicing the impartiality of the research reported.

## Funding

Funding was received from The Medical Research Council UK (MRC/Academy of Medical Sciences Clinician Scientist Fellowship Grant G0802796, to L F C, supporting T V N), Society for Endocrinology Early Career award to T V N, the Wellcome Trust (Grant No. 098051) to D W L, C L, E H W and for the M G P generation and phenotyping of the C57BL/6N background mice. R J G is supported by a Wellcome Clinical Research Training Fellowship (Grant No. Wbib92024MA). R L, D R, S O R and A P C are funded by the Medical Research Council (MRC) Metabolic Disease Unit (MRC_MC_UU_12012/1). L G was supported by Biotechnology and Biological Sciences Research Council (BBSRC), award BB/L00267/1 and Rosetrees Trust.

## References

[bib1] Anthonsen MW Ronnstrand L Wernstedt C Degerman E Holm C 1998 Identification of novel phosphorylation sites in hormone-sensitive lipase that are phosphorylated in response to isoproterenol and govern activation properties in vitro. Journal of Biological Chemistry 273 215–221. (10.1074/jbc.273.1.215) 9417067

[bib2] Asai M Ramachandrappa S Joachim M Shen Y Zhang R Nuthalapati N Ramanathan V Strochlic DE Ferket P Linhart K 2013 Loss of function of the melanocortin 2 receptor accessory protein 2 is associated with mammalian obesity. Science 341 275–278. (10.1126/science.1233000) 23869016 PMC3788688

[bib3] Balthasar N Dalgaard LT Lee CE Yu J Funahashi H Williams T Ferreira M Tang V McGovern RA Kenny CD 2005 Divergence of melanocortin pathways in the control of food intake and energy expenditure. Cell 123 493–505. (10.1016/j.cell.2005.08.035) 16269339

[bib4] Berwick DC Hers I Heesom KJ Moule SK Tavare JM 2002 The identification of ATP-citrate lyase as a protein kinase B (Akt) substrate in primary adipocytes. Journal of Biological Chemistry 277 33895–33900. (10.1074/jbc.M204681200) 12107176

[bib5] Braun TP Orwoll B Zhu X Levasseur PR Szumowski M Nguyen ML Bouxsein ML Klein RF Marks DL 2012 Regulation of lean mass, bone mass, and exercise tolerance by the central melanocortin system. PLoS ONE 7 e42183. 22848742 10.1371/journal.pone.0042183 PMC3407101

[bib6] Chan LF Webb TR Chung TT Meimaridou E Cooray SN Guasti L Chapple JP Egertova M Elphick MR Cheetham ME 2009 MRAP and MRAP2 are bidirectional regulators of the melanocortin receptor family. PNAS 106 6146–6151. (10.1073/pnas.0809918106) 19329486 PMC2661846

[bib7] Chen J Ingham N Clare S Raisen C Vancollie VE Ismail O McIntyre RE Tsang SH Mahajan VB Dougan G 2013 Mcph1-deficient mice reveal a role for MCPH1 in otitis media. PLoS ONE 8 e58156. 23516444 10.1371/journal.pone.0058156 PMC3596415

[bib8] Chypre M Zaidi N Smans K 2012 ATP-citrate lyase: a mini-review. Biochemical and Biophysical Research Communications 422 1–4. (10.1016/j.bbrc.2012.04.144) 22575446

[bib9] Cone RD 2005 Anatomy and regulation of the central melanocortin system. Nature Neuroscience 8 571–578. (10.1038/nn1455) 15856065

[bib10] De Marinis E Martini C Trentalance A Pallottini V 2008 Sex differences in hepatic regulation of cholesterol homeostasis. Journal of Endocrinology 198 635–643. (10.1677/JOE-08-0242) 18603607

[bib11] Egan JJ Greenberg AS Chang MK Wek SA Moos MC Jr Londos C 1992 Mechanism of hormone-stimulated lipolysis in adipocytes: translocation of hormone-sensitive lipase to the lipid storage droplet. PNAS 89 8537–8541. (10.1073/pnas.89.18.8537) 1528859 PMC49955

[bib12] Elia M Livesey G 1992 Energy expenditure and fuel selection in biological systems: the theory and practice of calculations based on indirect calorimetry and tracer methods. World Review of Nutrition and Dietetics 70 68–131. (10.1159/000421672) 1292242

[bib13] Flatt JP 1991 Assessment of daily and cumulative carbohydrate and fat balances in mice. Journal of Nutritional Biochemistry 2 193–202. (10.1016/0955-2863(91)90016-X)

[bib14] Franklin KBJ Paxinos G 2012 Paxinos and Franklin’s the Mouse Brain in Stereotaxic Coordinates. Amsterdam, The Netherlands: Academic Press.

[bib15] Gorrigan RJ Guasti L King P Clark AJ Chan LF 2011 Localisation of the melanocortin-2-receptor and its accessory proteins in the developing and adult adrenal gland. Journal of Molecular Endocrinology 46 227–232. (10.1530/JME-11-0011) 21367968 PMC3111094

[bib16] Holder JL Jr Zhang L Kublaoui BM DiLeone RJ Oz OK Bair CH Lee YH Zinn AR 2004 Sim1 gene dosage modulates the homeostatic feeding response to increased dietary fat in mice. American Journal of Physiology: Endocrinology and Metabolism 287 E105–E113. 14982752 10.1152/ajpendo.00446.2003

[bib17] Huszar D Lynch CA Fairchild-Huntress V Dunmore JH Fang Q Berkemeier LR Gu W Kesterson RA Boston BA Cone RD 1997 Targeted disruption of the melanocortin-4 receptor results in obesity in mice. Cell 88 131–141. (10.1016/S0092-8674(00)81865-6) 9019399

[bib18] Karp NA Melvin D Mott RF 2012 Robust and sensitive analysis of mouse knockout phenotypes. PLoS ONE 7 e52410. 23300663 10.1371/journal.pone.0052410 PMC3530558

[bib19] Kublaoui BM Holder JL Jr Gemelli T Zinn AR 2006a Sim1 haploinsufficiency impairs melanocortin-mediated anorexia and activation of paraventricular nucleus neurons. Molecular Endocrinology 20 2483–2492. 10.1210/me.2005-0483 16728530

[bib20] Kublaoui BM Holder JL Jr Tolson KP Gemelli T Zinn AR 2006b SIM1 overexpression partially rescues agouti yellow and diet-induced obesity by normalizing food intake. Endocrinology 147 4542–4549. 16709610 10.1210/en.2006-0453

[bib21] Kublaoui BM Gemelli T Tolson KP Wang Y Zinn AR 2008 Oxytocin deficiency mediates hyperphagic obesity of Sim1 haploinsufficient mice. Molecular Endocrinology 22 1723–1734. (10.1210/me.2008-0067) 18451093 PMC2453606

[bib22] Lee AK Mojtahed-Jaberi M Kyriakou T Astarloa EA Arno M Marshall NJ Brain SD O’Dell SD 2010 Effect of high-fat feeding on expression of genes controlling availability of dopamine in mouse hypothalamus. Nutrition 26 411–422. (10.1016/j.nut.2009.05.007) 19811894 PMC2839073

[bib23] Livak KJ Schmittgen TD 2001 Analysis of relative gene expression data using real-time quantitative PCR and the 2(-Delta Delta C(T)) method. Methods 25 402–408. (10.1006/meth.2001.1262) 11846609

[bib24] Marsh DJ Hollopeter G Huszar D Laufer R Yagaloff KA Fisher SL Burn P Palmiter RD 1999 Response of melanocortin-4 receptor-deficient mice to anorectic and orexigenic peptides. Nature Genetics 21 119–122. (10.1038/5070) 9916804

[bib25] McIntyre RE Lakshminarasimhan Chavali P Ismail O Carragher DM Sanchez-Andrade G Forment JV Fu B Del Castillo Velasco-Herrera M Edwards A van der Weyden L 2012 Disruption of mouse Cenpj, a regulator of centriole biogenesis, phenocopies Seckel syndrome. PLoS Genetics 8 e1003022. 23166506 10.1371/journal.pgen.1003022 PMC3499256

[bib26] Metherell LA Chapple JP Cooray S David A Becker C Ruschendorf F Naville D Begeot M Khoo B Nurnberg P 2005 Mutations in MRAP, encoding a new interacting partner of the ACTH receptor, cause familial glucocorticoid deficiency type 2. Nature Genetics 37 166–170. 15654338 10.1038/ng1501

[bib27] Michaud JL Rosenquist T May NR Fan CM 1998 Development of neuroendocrine lineages requires the bHLH-PAS transcription factor SIM1. Genes & Development 12 3264–3275. 9784500 10.1101/gad.12.20.3264 PMC317216

[bib28] Michaud JL Boucher F Melnyk A Gauthier F Goshu E Levy E Mitchell GA Himms-Hagen J Fan CM 2001 Sim1 haploinsufficiency causes hyperphagia, obesity and reduction of the paraventricular nucleus of the hypothalamus. Human Molecular Genetics 10 1465–1473. (10.1093/hmg/10.14.1465) 11448938

[bib29] Nogueiras R Wiedmer P Perez-Tilve D Veyrat-Durebex C Keogh JM Sutton GM Pfluger PT Castaneda TR Neschen S Hofmann SM 2007 The central melanocortin system directly controls peripheral lipid metabolism. Journal of Clinical Investigation 117 3475–3488. (10.1172/JCI31743) 17885689 PMC1978426

[bib30] Novoselova TV Jackson D Campbell DC Clark AJ Chan LF 2013 Melanocortin receptor accessory proteins in adrenal gland physiology and beyond. Journal of Endocrinology 217 R1–R11. 23418361 10.1530/JOE-12-0501

[bib31] Perez-Tilve D Hofmann SM Basford J Nogueiras R Pfluger PT Patterson JT Grant E Wilson-Perez HE Granholm NA Arnold M 2010 Melanocortin signaling in the CNS directly regulates circulating cholesterol. Nature Neuroscience 13 877–882. (10.1038/nn.2569) 20526334 PMC3100172

[bib32] Sebag JA Hinkle PM 2009 Opposite effects of the melanocortin-2 (MC2) receptor accessory protein MRAP on MC2 and MC5 receptor dimerization and trafficking. Journal of Biological Chemistry 284 22641–22648. (10.1074/jbc.M109.022400) 19535343 PMC2755671

[bib33] Sebag JA Hinkle PM 2010 Regulation of G protein-coupled receptor signaling: specific dominant-negative effects of melanocortin 2 receptor accessory protein 2. Science Signaling 3 ra28. 20371771 10.1126/scisignal.2000593 PMC2992810

[bib34] Sebag JA Zhang C Hinkle PM Bradshaw AM Cone RD 2013 Developmental control of the melanocortin-4 receptor by MRAP2 proteins in zebrafish. Science 341 278–281. (10.1126/science.1232995) 23869017 PMC4255277

[bib35] Shimomura I Shimano H Korn BS Bashmakov Y Horton JD 1998 Nuclear sterol regulatory element-binding proteins activate genes responsible for the entire program of unsaturated fatty acid biosynthesis in transgenic mouse liver. Journal of Biological Chemistry 273 35299–35306. (10.1074/jbc.273.52.35299) 9857071

[bib36] Skarnes WC Rosen B West AP Koutsourakis M Bushell W Iyer V Mujica AO Thomas M Harrow J Cox T 2011 A conditional knockout resource for the genome-wide study of mouse gene function. Nature 474 337–342. (10.1038/nature10163) 21677750 PMC3572410

[bib37] Speakman JR 2013 Measuring energy metabolism in the mouse – theoretical, practical, and analytical considerations. Frontiers in Physiology 4 34 (10.3389/fphys.2013.00034) 23504620 PMC3596737

[bib38] Tolson KP Gemelli T Gautron L Elmquist JK Zinn AR Kublaoui BM 2010 Postnatal Sim1 deficiency causes hyperphagic obesity and reduced Mc4r and oxytocin expression. Journal of Neuroscience 30 3803–3812. (10.1523/JNEUROSCI.5444-09.2010) 20220015 PMC3285557

[bib39] Tschop MH Speakman JR Arch JR Auwerx J Bruning JC Chan L Eckel RH Farese RV Jr Galgani JE Hambly C 2012 A guide to analysis of mouse energy metabolism. Nature Methods 9 57–63. 10.1038/nmeth.1806 PMC3654855 22205519

[bib40] Valassi E Scacchi M Cavagnini F 2008 Neuroendocrine control of food intake. Nutrition, Metabolism, and Cardiovascular Diseases 18 158–168. (10.1016/j.numecd.2007.06.004) 18061414

[bib41] White JK Gerdin AK Karp NA Ryder E Buljan M Bussell JN Salisbury J Clare S Ingham NJ Podrini C 2013 Genome-wide generation and systematic phenotyping of knockout mice reveals new roles for many genes. Cell 154 452–464. (10.1016/j.cell.2013.06.022) 23870131 PMC3717207

